# Carriage of *stx2a* Differentiates Clinical and Bovine-Biased Strains of *Escherichia coli* O157

**DOI:** 10.1371/journal.pone.0051572

**Published:** 2012-12-11

**Authors:** Smriti Shringi, Carrie Schmidt, Kaya Katherine, Kelly A. Brayton, Dale D. Hancock, Thomas E. Besser

**Affiliations:** 1 Department of Veterinary Microbiology and Pathology, Washington State University, Pullman, Washington, United States of America; 2 Department of Veterinary Clinical Sciences, Washington State University, Pullman, Washington, United States of America; U. S. Salinity Lab, United States of America

## Abstract

**Background:**

Shiga toxin (Stx) are cardinal virulence factors of enterohemorrhagic *E. coli* O157:H7 (EHEC O157). The gene content and genomic insertion sites of Stx-associated bacteriophages differentiate clinical genotypes of EHEC O157 (CG, typical of clinical isolates) from bovine-biased genotypes (BBG, rarely identified among clinical isolates). This project was designed to identify bacteriophage-mediated differences that may affect the virulence of CG and BBG.

**Methods:**

Stx-associated bacteriophage differences were identified by whole genome optical scans and characterized among >400 EHEC O157 clinical and cattle isolates by PCR.

**Results:**

Optical restriction maps of BBG strains consistently differed from those of CG strains only in the chromosomal insertion sites of Stx2-associated bacteriophages. Multiplex PCRs (*stx1, stx2a,* and *stx2c* as well as Stx-associated bacteriophage - chromosomal insertion site junctions) revealed four CG and three BBG that accounted for >90% of isolates. All BBG contained *stx2c* and Stx2c-associated bacteriophage – *sbcB* junctions. All CG contained *stx2a* and Stx2a-associated bacteriophage junctions in *wrbA* or *argW*.

**Conclusions:**

Presence or absence of *stx2a* (or another product encoded by the Stx2a-associated bacteriophage) is a parsimonious explanation for differential virulence of BBG and CG, as reflected in the distributions of these genotypes in humans and in the cattle reservoir.

## Introduction

Enterohemorrhagic *E. coli* O157:H7 (EHEC O157) is an important cause of food- and water-borne illnesses in developed nations. Diseases associated with EHEC O157 infections include hemorrhagic colitis (HC) and the hemolytic uremic syndrome (HUS) [1]. HUS following EHEC O157 infection is associated with 3% to 5% mortality [2]. Cattle are a major reservoir of EHEC O157 and consumption of contaminated bovine-origin foods is frequently linked to EHEC O157 outbreaks [3], [4].

Shiga toxin (Stx) expression is an important virulence factor of EHEC O157. Stx are encoded in late genes of lambdoid bacteriophages; specific bacteriophages, associated with specific Stx variants, are typically inserted at one or two preferred chromosomal locations. Bacteriophages play a major role in generating genetic diversity in the EHEC O157 genome [5], [6], [7], [8], [9], [10], [11], [12], [13]. Molecular epidemiological studies using Stx-associated bacteriophage insertion (SBI) sites for strain differentiation have shown that EHEC O157 can be classified into several genotypes [5], [10], [14]. Bovine-biased genotypes (BBG) are significantly over-represented among cattle isolates compared to clinical isolates, whereas clinical genotypes (CG) are typical of clinical isolates and are also frequently isolated from cattle [15], [16]. Other genotyping methods also demonstrate a similar bias in distribution of EHEC O157 lineages/clades among cattle and human host [6], [8], [12]. Both BBG and CG usually carry both EHEC O157 cardinal virulence factors: production of one or more Stx and expression of the Locus of Enterocyte Effacement (LEE) pathogenicity island required for intestinal colonization [1], [17]. The pronounced differences in their frequency of isolation from human disease and from cattle suggest that BBG may have reduced virulence. This possibility was supported by the recent demonstration that CG strains cause more severe clinical signs, more severe histopathologic lesions, and higher mortality than BBG strains in two animal models of human disease [15].

We hypothesize that specific bacteriophage-associated genetic factors underlie the differential virulence of CG and BBG EHEC O157. To test this hypothesis, we: 1) identified consistent bacteriophage-mediated genetic differences associated with four BBG and three CG strains using optical mapping; 2) developed a 12-plex PCR to efficiently SBI genotype EHEC O157 isolates and formatted a biologically relevant SBI genotype nomenclature; and 3) applied SBI typing to evaluate the consistency of the genetic differences discovered in the optically mapped strains in approximately 200 additional isolates each from cattle and human clinical sources. The presence of specific *stx2* variants and/or their associated bacteriophages were strongly associated with specific SBI genotypes and with their relative frequency of isolation from human illness and cattle reservoir.

## Materials and Methods

### Bacterial Strains

For optical mapping, seven EHEC O157 strains representing the four most common SBI genotypes (one isolate CG-1 and two isolates each CG-3, BBG-5 and BBG-6) isolated from cattle and banked at the Field Disease Investigation Unit at Washington State University [5] were selected by random number table (Table 1). An additional larger set of EHEC O157 strains were randomly selected from the strain bank, stratified to include no more than one isolate per cattle farm per calendar year, for comparison with the optically mapped strains. The bovine isolates included 227 EHEC O157 isolates from four countries including Canada, Japan, Scotland and several states within the USA (WA, OR, ID, CO, AZ, TX, KS, MN, SD, IA, NE, PA, and NY) obtained in field research studies conducted between 1991 and 2010. Human isolates (n = 192), kindly provided by the Washington State Department of Health, included 40–45 isolates randomly selected from among reported cases for each year from 2006 through 2009, and all 19 isolates available from 2010 at the time this study was initiated. Each isolate was confirmed as EHEC O157 by PCR detection of *eae* and *flic_H7_* by PCR [18], [19], [20] and latex agglutination detection of O157 antigen (*E. coli* PRO O157, Hardy Diagnostics, USA).

**Table 1:** Summary of genetic differences among three clinical genotypes (CG) and four bovine-biased genotypes (BBG) of EHEC O157 identified using optical mapping technology.

				Total number of genetic rearrangements^c^			
Strain	SBI Genotype^a^	Genome size (bp)^b^	BamHI fragments (No.)	Insertions	Deletions	RFLP	Others
E2325	CG-1 (AY2)	5,407,641	478	2	6	24	1 Inv, 3 Sub
E5252	CG-3 (WY12)	5,376,952	488	0	7	7	1 Sub
E3046	CG-3 (WY12)	5,486,716	540	1	0	16	1 Inv, 1 Sub
E5880	BBG-5 (SY2c)	5,426,915	532	2	7	12	2 Inv, 5 Sub
E3855	BBG-5 (SY2c)	5,390,209	500	5	10	16	3 Sub
E2309	BBG-6 (ASY12c)	5,504,252	518	3	6	11	1 Inv, 1 Tnv, 3 Sub
E6996	BBG-6 (ASY12c)	5,583,835	551	4	5	18	4 Sub

a Modified SBI genotype for each isolate is indicated in parenthesis. This shows the concatenation of presence of specific bacteriophage at *argW*, *sbcB*, *wrbA* or *yehV* locations designated by uppercase first letter of each insertion site (A, S, W or Y,) followed by the presence of *stx1*, *stx2a* or *stx2c* denoted by 1,2 or 2c.

b Genome sizes predicted on the basis of optical maps of each strain.

c Number of genetic rearrangements in comparison with strain Sakai (CG-3). Insertions = presence of additional restriction fragments; deletions = loss of RE fragments; RFLPs (restriction fragment length polymorphisms) = altered sizes and/or numbers of RE fragments; inversions (Inv) = RE fragments in reversed order; substitutions (Sub) = altered RE fragments; transversion (Tnv) = RE fragments in reversed order and in a different location.

### Optical Mapping

Optical maps of seven EHEC O157 isolates were constructed and provided by OpGen technologies, Inc (Madison, WI, USA) using the procedure described previously [21], [22]. We compared the whole genome optical maps of each strain with the *in silico Bam*HI restriction enzyme (RE) map of strain Sakai (BA000007) using Map Viewer software (OpGen). An inventory of genomic differences involving restriction fragments >2 kb (the resolution of the optical mapping technology) was prepared for each strain in comparison to the reference Sakai sequence[22].

### Multiplex PCR Method for SBI Genotyping

To confirm the genetic differences between CG and BBG strains detected by optical mapping, a multiplex PCR was developed and standardized (Table 2). The PCR targets included were the left and right bacteriophage-bacterial backbone junctions of three different bacteriophages potentially inserted at four sites (*yehV*, *wrbA*, *argW* and *sbcB*) in the EHEC O157 genome, a previously identified variable bacteriophage-*yehV* right junction [5], [10], and *stx1, stx2a,* and *stx2c*. Each of the forward primers was 5′-labeled with a fluorescent dye (PET, 6-FAM VIC or NED) as indicated (Table 2). This multiplex PCR is capable of amplifying 16 different genetic regions; the twelve listed above and in addition the four intact chromosomal insertion sites.

**Table 2:** Oligonucleotide primers and amplified PCR products in the study.

Primer	Dye	Oligonucleotide sequences (5′-3′)	Target	Amplicon (bp)	Reference
2851-80	PET	GTGCTTGGGTCTTTTCTCTG	Phage-*sbcB* left junction	914	[26]
*sbcB*-R2	None	TCCAGGCGTAAGGATCGTAG			This study
*sbcB*-F	VIC	GACAGCAGAAACAACGGATTTAAC	Phage-*sbcB* right junction	730	This study
2851-82	None	CCAGCGTGGGATAAAAGAGAATC			[26]
*sbcB*-F	VIC	GACAGCAGAAACAACGGATTTAAC	*sbcB* insertion site	406	This study
*sbcB*-R2	None	TCCAGGCGTAAGGATCGTAG			This study
F	6FAM	CACCGGAAGGACAATTCATC	Phage-*yehV* left junction	824	[10]
B	None	AACAGATGTGTGGTGAGTGTCTG			[10]
A	PET	AAGTGGCGTTGCTTTGTGAT	Phage-*yehV* right junction	702	[10]
E	None	GATGCACAATAGGCACTACGC			[10]
A	PET	AAGTGGCGTTGCTTTGTGAT	Phage-*yehV* variant right junction	295	[10]
E3	None	AGCGATACAGATCTCAACAC			This study
A	PET	AAGTGGCGTTGCTTTGTGAT	*yehV* insertion site	340	[10]
B	None	AACAGATGTGTGGTGAGTGTCTG			[10]
C	6FAM	AGGAAGGTACGCATTTGACC	Phage-*wrbA* right junction	592	[10]
G	None	ATCGTTCGCAAGAATCACAA			[10]
H	PET	CCGACCTTTGTACGGATGTAA	Phage-*wrbA* left junction	506/537	[10]
D	None	CGAATCGCTACGGAATAGAGA			[10]
C	6FAM	AGGAAGGTACGCATTTGACC	*wrbA* insertion site	314	[10]
D	None	CGAATCGCTACGGAATAGAGA			[10]
*argW*-A	VIC	CCGTAACGACATGAGCAACAAG	Phage-*argW* right junction	583	This study
*argW*-B	None	GCAGTATCACGCAGAGCTGAAG			This study
*argW*-C	6FAM	GCATCTCACCGACGATAACA	Phage-*argW* left junction	462	This study
*argW*-D	None	AATTAGCCCTTAGGAGGGGC			This study
*argW*-A	VIC	CCGTAACGACATGAGCAACAAG	*argW* insertion site	216	This study
*argW*-D	None	AATTAGCCCTTAGGAGGGGC			This study
Stx1-2	VIC	CACAGACTGCTGCAGTGAGG	Stx1	475	[20]
Stx1-1	None	CAGTTAATGTGGTGGCGAAG			[20]
stx2-1072F	NED	AGGATGACACATTTACAGTGAAGGTT	Stx2a -specific	126	[50]
stx2-1197R	None	CACAGGTACTGGATTTGATTGTGAC			[50]
stx2c-F858	NED	CGACAGGCCCGTTATAAAAA	Stx2c -specific	243	This study
stx2c-R1100	None	GGCCACTTTTACTGTGAATGTATC			[5]

The PCR reaction mixture included 1X PCR buffer, 0.05 U/ul of Platinum Taq DNA Polymerase (Invitrogen, USA), 2.4 mM MgCl_2_, 0.4 mM dNTP, 0.2 µM of each forward and reverse primers to amplify the junctions of 4 bacteriophages (A, B, C, D, E, F, G, H, *argW*-A, *argW*-B, *argW*-C, *argW*-D, *sbcB*-F, *sbcB*-R2, 2851-80, and 2851-82), 0.3 µM of each *stx1*-2 and *stx1*-1 to amplify *stx1*, 0.2 µM of each *stx2*-1072F and *stx2*-1197R to amplify *stx2a*, 0.1 µM of each *stx2c*-F858 and *stx2c*-R1100 to amplify *stx2c*, 0.12 µM of primer E3, and 1 µl of Chelex DNA extract supernatant as template. For DNA extraction, a colony of bacteria from a Columbia blood agar (Hardy diagnostics) was added and mixed with 200 µl of 5% Chelex 100 Resin (Bio-Rad), boiled for 10 min, and centrifuged at 13,000 rpm for 1 min. The PCR cycling conditions included initial denaturation at 95°C for 5 min followed by 35 cycles of 94°C for 30 sec, 58°C for 45 sec, and 72°C for 1 min 30 sec and a final extension at 72°C for 10 min. Strains E5880 (BBG-5), E10970 (CG-1) and Sakai (CG-3) were used as positive controls.

The method used to differentiate *stx2a* and *stx2c* in the current study did not differentiate *stx2c* and *stx2d*-activatable. Therefore, we further tested all *stx2c* strains (n = 131) to detect possible presence of *stx2d*-activatable using one or both of the two subtyping methods [23], [24]. These analyses confirmed that no strains in this study carried *stx2d*-activatable. Additionally, two of the *stx2c* carrying strains, one each from human and cattle sources, carried an insertion sequence within *stx2c* coding region as demonstrated by partial DNA sequencing (data not shown).

### Capillary Electrophoresis

For capillary electrophoresis, 2 µl of PCR product was mixed with 12.5 µl of Hi-Di formamide (Applied Biosystems, Foster City, CA) and 0.5 µl of Liz 1200 size standard (Applied Biosystems). The capillary electrophoresis was performed using ABI-3730 DNA analyzer at WSU Genomic Core. GeneMarker software (SoftGenetics, LLC, State College, PA, USA 16803) was used to identify the electropherogram peaks corresponding to each PCR product according to their molecular size and associated dye colors.

### Statistical Analysis

Association of the Stx-associated bacteriophage and *stx2* gene variant content with the bovine-biased and clinical genotypes of EHEC O157 was analyzed using the Fisher’s exact test.

## Results

### Optical Mapping

A total of 112 polymorphisms were identified in the optical scan restriction maps of seven representative SBI genotype strains compared to the *in silico Bam*HI restriction map of the genome sequence of Sakai (Table 1 and Table S1). Of these polymorphisms, 32 unique to one or more CG strains (and therefore uninformative about BBG - CG strain differences) were not further investigated. Of remaining 80 polymorphisms, 30 affected one or more strains belonging to both CG and BBG, while 50 were unique to one or more BBG strains.

Only one polymorphism differentiated all BBG strains from all CG strains, an insertion detected in BBG strains but absent in CG strains (Table 3). This ∼60 kbp insertion affected *Bam*HI RE fragments 334–335 of Sakai strain (2,740,136–2,803,536 bp), a region that includes *sbcB,* an alternative Stx2c-associated bacteriophage insertion site, suggesting the presence of this phage in BBG strains [9], [25], [26]. This was confirmed when PCR (Table 2) on the optically mapped strains amplified Stx2c-associated bacteriophage sequences adjacent to *sbcB* sequences in all four BBG strains (Table 3 and Figure S1). In contrast, optically mapped CG strains lacked this insertion and amplified PCR products consistent with an intact *sbcB* locus.

**Table 3:** Selected BBG polymorphisms in comparison to reference strain Sakai (CG-3).

Sakai strain^a^							
Start Location (bp)	End Location (bp)	Fragment	Features^b^	BBG strains^c^	Polymorphism	Size (bp)	Stx-associated bacteriophage^d^
2,740,136	2,803,536	334–335	SpLE2 (*sbcB*)	1,2,3,4	Insertion	∼60,000	Stx2c
3,172,859	3,198,717	377–380	Sp16 (*argW*)	3	Insertion	48,365	Stx2a
3,198,717	3,219,331	381–382	SP16 (*argW*)	4	Insertion	58,185	Stx2a
1,246,750	1,310,488	142–151	Sp5	1,2,3,4	Deletion	63,738	Stx2a
2,912,120	2,943,884	347–349	Sp15	1	RFLP	3,718	Stx1
2,912,120	2,943,884	347–349	Sp15	2	Substitution	13,840	Stx1

a Sakai strain (CG-3) chromosomal location, *in silico Bam*HI fragments, and features present in the polymorphic region.

b Genes cited are known Stx-associated bacteriophage insertion sites. In Sakai, Sp5 is the Stx2-associated bacteriophage inserted in *wrbA* and Sp15 is the Stx1-associated bacteriophage inserted in *yehV*; Sp = Sakai prophage; SpLE = Sakai prophage like elements.

c BBG isolate(s) in which the polymorphism was detected; 1: E5880; 2: E3855; 3: E2039; 4: E6996.

d Stx-associated bacteriophage potentially involved in polymorphism based on optical maps.

We also evaluated all other reported alternative insertion sites for Stx-associated bacteriophages (i.e., within or adjacent to *wrbA*, *yehV*, *argW*, *ssrA*, *yecE*, Z2755, *intS*, *yfbO*, *sgcA*, and *nrdA-yfaL*) in the optical maps of the tested strains [9], [25], [26], [27], [28], [29], [30], [31], [32]. Three of these loci (*wrbA*, *yehV* and *argW*) also revealed indels suggestive of bacteriophage insertion or deletion (Table 3). The insertion of Stx2-associated bacteriophage sequences within *argW* was confirmed in both of the BBG-6 strains and in the CG-1 strain by PCR (Table 3 and Figure S2). The remaining candidate insertions in *wrbA* and *yehV* were previously recognized and have been utilized in SBI genotyping [5], [10] (Table 3).

Corresponding to the Stx-associated bacteriophage differences detected in optically mapped strains, *stx2a* was detected by PCR in all CG-1 and CG-3 strains, whereas *stx2c* (but not *stx2a*) was detected in all BBG-5 and BBG-6 strains. The absence of *stx2a* in BBG-6 strains that contain Stx2-associated bacteriophage sequences within *argW* was associated with altered *Bam*HI restriction fragments consistent with a partial Stx2a-associated bacteriophage at this locus (Figure S2): E2325 (CG1, with an intact *stx2a* encoding bacteriophage) has four restriction fragments totaling 59,809 bp, whereas BBG-6 strain E6996 has seven restriction fragments totaling 58,185 bp and BBG-6 strain E2309 has five restriction fragments totaling 50,780 bp.

### Modified SBI Genotype Designations

Since the multiplex PCR described here includes additional targets compared to previous SBI genotyping reports [5], [10], [14] modification of the previously used SBI genotype nomenclature was necessary. In place of the arbitrary numerical genotypes previously used (e.g., CG-1 and BBG-5), genotypes are identified here by concatenation of the upper case letters of bacteriophage insertion loci (A = *argW,* S = *sbcB*, W = *wrbA,* and Y = *yehV*), followed by numerical indication of the Stx variants detected (i.e., 1, 2, and/or 2c, Table 4). Bacteriophage insertion loci were considered occupied if either left or right (or both) bacteriophage – bacterial backbone junctions were amplified whereas these had been considered distinct genotypes in the previous system. As a result, the modified nomenclature resulted in both splitting and lumping of genotypes in comparison with the former system (Table 4). Isolates formerly designated CG-1 (n = 91) now include AY2 (61.5%), ASY22c (18.7%), ASY2 (14.3%) and SY2c (5.5%) genotypes. Isolates formerly designated CG-3 (n = 173) remain, with a single exception, a single genotype, WY12. Isolates formerly designated BBG-5 (n = 46) now include SY2c (89%), ASY2c (4.4%) and ASY22c (6.5%). Isolates formerly designated BBG-6 (n = 60) now include genotypes ASY12c (76.6%), SY12c (21.7%), and SY122c (1.7%). Finally, all isolates formerly designated BBG-7 are now WY12. Under this modified SBI genotype nomenclature, the optically mapped strains represent the modal genotypes of the former SBI designations (Table 1): CG-1 strain E2325 is AY2, CG-3 strains E5252 and E3046 are both WY2, BBG-5 strains E5880 and E3855 are both SY2c, and BBG-6 strains E2309 and E6996 are both ASY12c.

**Table 4:** SBI Genotyping on human and bovine isolates of EHEC O157.

SBI Genotype Coding^a^	Bovine (%)	Human (%)	Total (%)	SBI Genogroup^b^	P-value^b^	Former SBI genotype designation (%)^c^
WY12	92 (40.5)	97 (50.5)	189 (45.1)	CG	0.048	3 (91), 7 (6.4), 18 (2.1), 22 (0.5)
AY2	11 (4.8)	47 (24.5)	58 (13.8)	CG	<0.0001	1 (96.6), 19 (3.5)
SY2c	45 (19.8)	2 (1.0)	47 (11.2)	BBG	<0.0001	5 (87.2), 1 (10.6), 19 (2.1)
ASY12c	38 (16.7)	8 (4.2)	46 (11.0)	BBG	<0.0001	6 (100)
ASY22c	5 (2.2)	15 (7.8)	20 (4.8)	CG	0.01	1 (85), 5 (15)
SY12c	14 (6.2)	0 (0.0)	14 (3.3)	BBG	0.0004	6 (92.9), 16 (7.1)
ASY2	1 (0.4)	12 (6.3)	13 (3.1)	CG	0.0005	1 (100)
W2	3 (1.3)	8 (4.2)	11 (2.6)	Unclassified	0.12	4 (100)
WY2	4 (1.8)	0 (0.0)	4 (1.0)	Unclassified	N/A	2 (100)
Y	4 (1.8)	0 (0.0)	4 (1.0)	Unclassified	N/A	17 (75), 10 (25)
ASY2c	2 (0.9)	0 (0.0)	2 (0.5)	Unclassified	N/A	5 (100)
AWY12	1 (0.4)	1 (0.5)	2(0.5)	Unclassified	N/A	3, 18
AY	1 (0.4)	0 (0.0)	1 (0.2)	Unclassified	N/A	10 (100)
Y1	0 (0.0)	1 (0.5)	1 (0.2)	Unclassified	N/A	8 (100)
ASY1	1 (0.4)	0 (0.0)	1 (0.2)	Unclassified	N/A	13 (100)
AY1	1 (0.4)	0 (0.0)	1 (0.2)	Unclassified	N/A	13 (100)
WY1	1 (0.4)	0 (0.0)	1 (0.2)	Unclassified	N/A	22 (100)
SWY2c	1 (0.4)	0 (0.0)	1 (0.2)	Unclassified	N/A	12 (100)
	0 (0.0)	1 (0.5)	1 (0.2)	Unclassified	N/A	9 (100)
SY122c	1 (0.4)	0 (0.0)	1 (0.2)	Unclassified	N/A	6 (100)
AWY2	1 (0.4)	0 (0.0)	1 (0.2)	Unclassified	N/A	2 (100)
Total	227	192	419			

a Concatenation of presence of specific bacteriophage at *argW*, *sbcB*, *wrbA* or *yehV* locations designated by uppercase first letter of each insertion site (A, S, W or Y,) followed by the presence of *stx1*, *stx2a* or *stx2c* denoted by 1,2 or 2c.

b The SBI Genotypes that are significantly over-represented among cattle isolates as compared to human isolates are referred as bovine-biased genotypes (BBG); the SBI Genotypes that show significantly over-representation/similar-representation among human isolates as compared to cattle isolates are referred as clinical genotypes (CG); the SBI genotypes that could not be evaluated for a bovine association due to small numbers (1–4 isolates) are designated “unclassified” genotypes in this manuscript (Fisher’s exact test P≤0.05).

c Percent of isolates belonging to most common previously defined SBI genotype [5], [10], [14].

### SBI Genotyping on Additional EHEC O157 Isolates

The distribution of SBI genotypes among cattle and human was determined by testing 419 additional EHEC O157 isolates. The PCR differentiated these isolates into 21 different genotypes (Table 4) of which eight genotypes represented a large majority (95%) of the isolates. Seven of these eight genotypes were significantly differentially represented in cattle and human hosts (P<0.05, Table 4). Three genotypes (SY2c, ASY12c and SY12c, n = 107) were significantly more frequent among bovine isolates (BBG, 42.7% of bovine isolates *vs* 5.2% of clinical isolates). Four SBI genotypes (WY12, AY2, ASY22c, and ASY2, n = 280) were significantly more frequent among clinical isolates (CG, 89.1% of clinical isolates *vs* 48% of bovine isolates). The remaining genotypes, not detected at significantly different frequencies from cattle and human sources, included W2 (11 isolates, including 8 clinical and 3 bovine) and thirteen additional SBI genotypes (n = 21 total). These SBI genotypes are designated “unclassified” genotypes in this manuscript. The 192 human isolates cumulatively belonged to ten genotypes: CG (89.1%), BBG (5.2%), and unclassified (5.7%). The 227 bovine isolates cumulatively belonged to 19 genotypes: CG (48%), BBG (42.7%), and unclassified (9.3%) (Table 4).

### Distribution of Stx2 Gene and Associated Bacteriophages

Stx2a-associated bacteriophage sequences detected adjacent to either *wrbA* and/or *argW* and the detection of *stx2a* were significantly associated with CG compared to BBG strains (*P*≤0.001): all CG have Stx2a-associated bacteriophage sequences inserted in *wrbA* and/or *argW* and carry *stx2a.* In contrast, the presence of Stx2c-associated bacteriophage sequences inserted in *sbcB* and the presence of *stx2c* were significantly more common in BBG compared to CG isolates (*P*≤0.001): all BBG had both these traits, while only 11.8% of CG belonged to genotypes (ASY2 and ASY22c) with Stx2c-associated bacteriophage sequences inserted in *sbcB,* and only 7.1% of CG isolates (ASY22c) carried *stx2c* (Table 5). Many BBG (42.9%) had Stx2a-associated bacteriophage sequences adjacent to *wrbA* or *argW* but lacked detectable *stx2a* (Table 5).

**Table 5:** Consistency of type of Stx-associated bacteriophage and *stx* in SBI genotypes in clinical and bovine strains.

			SBI genotypes^a^			
Stx-associated bacteriophage	Insertion site	Origin	CG	BBG	Unclassified^b^	Total
Stx2a	*wrbA* c	Bovine	92 (92)	0 (0)	11 (9)	103 (101)
		Human	97 (97)	0 (0)	9 (9)	106 (106)
	*argW* d	Bovine	17 (17)	38 (0)	7 (2)	62 (19)
		Human	74 (74)	8 (0)	1 (1)	83 (75)
Stx2c	*sbcB* d	Bovine	6 (5)	97 (97)	5 (4)	108 (106)
		Human	27 (15)	10 (10)	0 (0)	37 (25)
Stx1	*yehV* c	Bovine	109 (92)	97 (52)	18 (5)	224 (149)
		Human	171 (97)	10 (8)	2 (2)	183 (107)
Strains in study		Bovine	110	97	20	227
		Human	171	10	11	192

a Numbers of isolates with the corresponding Stx-associated bacteriophage sequences in the insertion site. Numbers in parentheses indicate the isolate also positive for the expected *stx*. Association of the Stx-associated bacteriophage and *stx2* gene variant with the BBG and CG strains was analyzed using the Fisher’s exact test (P≤0.05).

b Two strains of AWY12 and single strain of AWY2 had Stx2-associated bacteriophages inserted into both *argW* and *wrbA*, and our data cannot determine whether *stx2a* is carried by one or both of these bacteriophages.

c Stx-associated bacteriophage differences among SBI genotypes identified in earlier studies [5], [10].

d Stx-associated bacteriophage differences among SBI genotypes identified in this study.

Corresponding differences were observed in the frequency of carriage of *stx2* variants in human- and bovine-origin isolates. A large majority (85.9%) of the 192 human-origin isolates carried *stx2a* only and an additional 7.8% carried both *stx2a* and *stx2c,* while only 5.2% carried *stx2c* only and 1.0% carried neither *stx2* variant. Of 227 bovine isolates 49.8% carried *stx2a* only, 44.1% carried *stx2c* only, 2.6% carried both, and 3.5% carried neither variant.

## Discussion

Optical mapping technology accurately identified genetic changes consistent with bacteriophage insertions or deletions in EHEC O157 genotypes. This technology was used previously by Kotewicz et al. to identify differences in EHEC O157 strains representing SNP clusters and spinach outbreak strains [22], [30]. While Kotewicz et al. provided valuable information on genetic diversity among EHEC O157 strains of clinical origin; their clinical isolate strain set lacked defined BBG isolates. Our study was specifically designed to identify bacteriophage-related differences between the predominant CG (CG-1 and CG-3, now AY2 and WY12) and BBG (BBG-5 and BBG-6, now SY2c and ASY12c) groups that previously had been associated with differential virulence potential [15].

SBI has proven to be a useful genotyping technique to identify strains of EHEC O157 differing in distribution [5], [10], [14], gene expression [16] and virulence [15]. Although based on the content and insertional locations of bacteriophages (i.e., mobile genetic elements), SBI genotypes are empirically stable characteristics of EHEC O157 strains, as recently confirmed by Bono et al. [33] in their demonstration of close correlation between SBI genotypes and chromosomal backbone SNP-defined lineages of EHEC O157. Specifically, CG-1 (referred in current paper as AY2/ASY2/ASY22c) and CG-3 (WY12) correspond to SNP lineage II and I, respectively, and BBG-5 (SY2c) and BBG-6 (SY12c/ASY12c) corresponds to lineages III, IV, V and VII [33]. Therefore, the SBI multiplex PCR described here provides a means for presumptive identification of EHEC O157 lineages as defined by SNP typing.

SBI genotype designations were modified to incorporate data from additional targets and to provide a biologically-based nomenclature. The additional targets include independent detection of *stx2a* and *stx2c* variants and two additional chromosomal - bacteriophage insertion sites, *argW* and *sbcB.* Even before addition of these targets well over 20 different SBI genotypes had been reported, and this cumbersome number as well as the arbitrary nature of the ordinal genotype designations threatened the usability of the system. Therefore, here we propose identification of SBI genotypes by the concatenation of letters representing specific chromosomal insertion sites followed by numbers indicating the *stx* variants detected. Isolates classified by this revised SBI designation system are more strongly associated with specific hosts, suggesting that the system is consistent with the biological behavior of the bacterium.

The findings of this study clarify the bacteriophage related genetic differences characterizing BBG and CG, and confirm the strong association of *stx2* subtypes in these two genogroups.

Using a panel of 419 EHEC O157 geographically and temporally diverse cattle- and human-origin isolates, consistent patterns of Stx-associated bacteriophage insertions and *stx2* variant carriage defined by the optically mapped strains was consistently observed: CG strains were characterized by the presence of the Stx2a-associated bacteriophage sequences inserted into *argW* or *wrbA* and by carriage of *stx2a,* while BBG were characterized by Stx2c-associated bacteriophage sequences adjacent to *sbcB* and carriage of *stx2c*. Some CG strains additionally have Stx2c-associated bacteriophage sequences adjacent to *sbcB*, with or without *stx2c* carriage, and about half of BBG isolates additionally have Stx2a-associated bacteriophage sequences adjacent to *argW* but lack *stx2a*. The close association of *stx2a* with the more clinically relevant CG strains is the most striking finding of our study.

We recently demonstrated that CG strains (carrying *stx2a* with or without *stx2c*) induced more severe clinical symptoms, earlier and higher mortality, and more severe histopathological lesions compared to BBG strains (carrying *stx2c* only) in two animal models [15]. That study also demonstrated that CG strains produced higher amounts of ELISA-reactive Stx than BBG strains. A recent microarray study [16] demonstrated differential expression profiles of BBG and CG strains suggesting that the increased prevalence of EHEC O157 illness caused by certain strains is associated with over-expression of several virulence factors including *ehx,* genes on the LEE pathogenicity island, and pO157 genes, although no difference in *stx* expression was identified.

Other comparative genomic studies on EHEC O157 have demonstrated similar variation in the frequency of *stx* variants among cattle and human isolates. Using octamer-based genome scanning and LSPA-6 genotyping [12], [13], [34], EHEC O157 isolates were differentiated into three lineages: I, I/II, and II. Recent studies demonstrated concordance between LSPA-6 and SBI genotyping, indicating that the lineage I, I/II, II strains predominantly belonged to SBI genotypes CG-3, CG-1 and BBG-6, respectively [35], [36]. Ziebell et al. [37] and Zhang et al. [34] demonstrated that nearly all lineage II strains carried *stx*2c, nearly all of the lineage I strains carried *stx2a* and 50.0% of lineage I/II strains carried *stx*2c. Similarly a recent study on Japanese isolates also suggests *stx2a* is the most distinctive feature in clinical isolates compared to cattle isolates. The human isolates, predominantly LSPA-6 lineage I or I/II, typically carried *stx2a* alone or in combination with *stx1* or *stx2c* while LSPA-6 lineage II cattle isolates carried *stx2c* alone or in combination with *stx1* [38]. These investigators also demonstrated that lineage I (*stx1*-*stx2*) isolates showed multiple stress resistances when compared to the lineage II (*stx1*-*stx2c*) genotypes [39].

Other studies have evaluated association of *stx2a* and *stx2c* content with human virulence [23], [40], [41], [42], [43], [44], [45], [46], [47], [48], [49]. Several of these epidemiological studies [41], [42], [43], [45], [46], [47], [48] demonstrated that EHEC O157 carrying *stx2a* (with or without *stx2c*) rather than *stx2c* were more frequent among clinical isolates, in agreement with the results presented here. Recently, Kawano et al. [48] along with others [43], [45], [46] suggested that EHEC O157 carrying *stx2c* were associated with asymptomatic individuals and mild disease however it is also isolated from patients with severe disease (bloody diarrhea, HC and HUS) as shown by others [23], [40], [41], [44], [47], [49]. Furthermore, Fuller et al. recently demonstrated that purified Stx2a is more potent than Stx2c against primary human kidney cell lines and in mouse models [42]. It is possible that carriage and expression of *stx2a* alone is sufficient to confer increased virulence in animal models and increased expression of human disease, but alternatively these phenotypes may result in whole or in part on other genetic factors that are correlated with *stx2a*. Knock-out and complementation studies to evaluate the role of *stx2a* in otherwise isogenic strains may be required to clarify this question.

In conclusion, optical scanning proved effective at identifying large genetic indels associated with insertion or deletion of Stx-associated bacteriophages among major SBI genotypes of EHEC O157. The multiplex PCR described in this study provides a simple and rapid method to efficiently determine SBI genotypes of EHEC O157 isolates, now known to correlate with lineages within the EHEC O157 clade. We conclude that differential virulence of EHEC O157 genotypes may be due to variation in the presence of *stx2* variants and/or bacteriophages associated these variants. Unraveling the roles of these genetic elements in EHEC O157 virulence will improve our understanding of pathogenesis and may facilitate development of strategies for the prevention and control of this important food-borne pathogen.

## Supporting Information

Figure S1 **Differences in insertion of Stx2c-associated bacteriophage in** ***sbcB*** **.** The differences in insertion of Stx2c-associated bacteriophage in *sbcB* are shown by the hatched fragments. The yellow arrows indicate the phages (Sp) or prophage–like elements (SpLE) in sequenced strain Sakai (names shown below the map). Red marks indicate the insertion sites (*sbcB* and *yegQ*) for Stx-associated bacteriophage, and left junction (YL) for Stx1-associated bacteriophage inserted in *yehV* (names shown above the map). The restriction enzyme map of sequenced strain EC4115 (GenBank accession # CP001164) shows the known insertion of Stx2c-associated bacteriophage in *sbcB* locus (hatched fragments). The restriction enzyme map for Sakai and EC4115 are *in silico* maps and the other seven maps are optical maps of test strains. Plus (+) and minus (−) signs represent presence and absence of *stx* gene in the strains.
(TIF)

Figure S2 **Differences in insertion of Stx2a-associated bacteriophage in** ***argW*** **.** The differences in insertion of Stx2a-associated bacteriophage in *argW* are shown by the hatched fragments. The yellow arrows indicate the phages (Sp) in sequenced strain Sakai (names shown below the map). Red marks indicate the insertion sites (*argW*/*IntS)* for Stx-associated bacteriophage (names shown above the map). The restriction enzyme map of the sequenced strain EC4115 (GenBank accession # CP001164) shows the known insertion Stx2a-associated bacteriophage in *argW* locus (hatched fragments). The restriction enzyme map for Sakai and EC4115 are *in silico* maps and the other seven maps are optical maps of test strains. Plus (+) and minus (−) signs represent presence and absence of *stx* gene in the strains.
(TIF)

Table S1 **Polymorphic sites in optical maps of seven EHEC O157 strains when compared to the** ***in silico Bam*** **HI restriction enzyme map of the EHEC O157 Sakai strain (CG-3).**
(DOCX)
