# Supplementary figures and images for: Carriage of stx2a Differentiates Clinical and Bovine-Biased Strains of Escherichia coli O157
Source: PLoS One. 2012 Dec 11;7(12):e51572. doi: 10.1371/journal.pone.0051572 (PMC3519850; doi:10.1371/journal.pone.0051572)

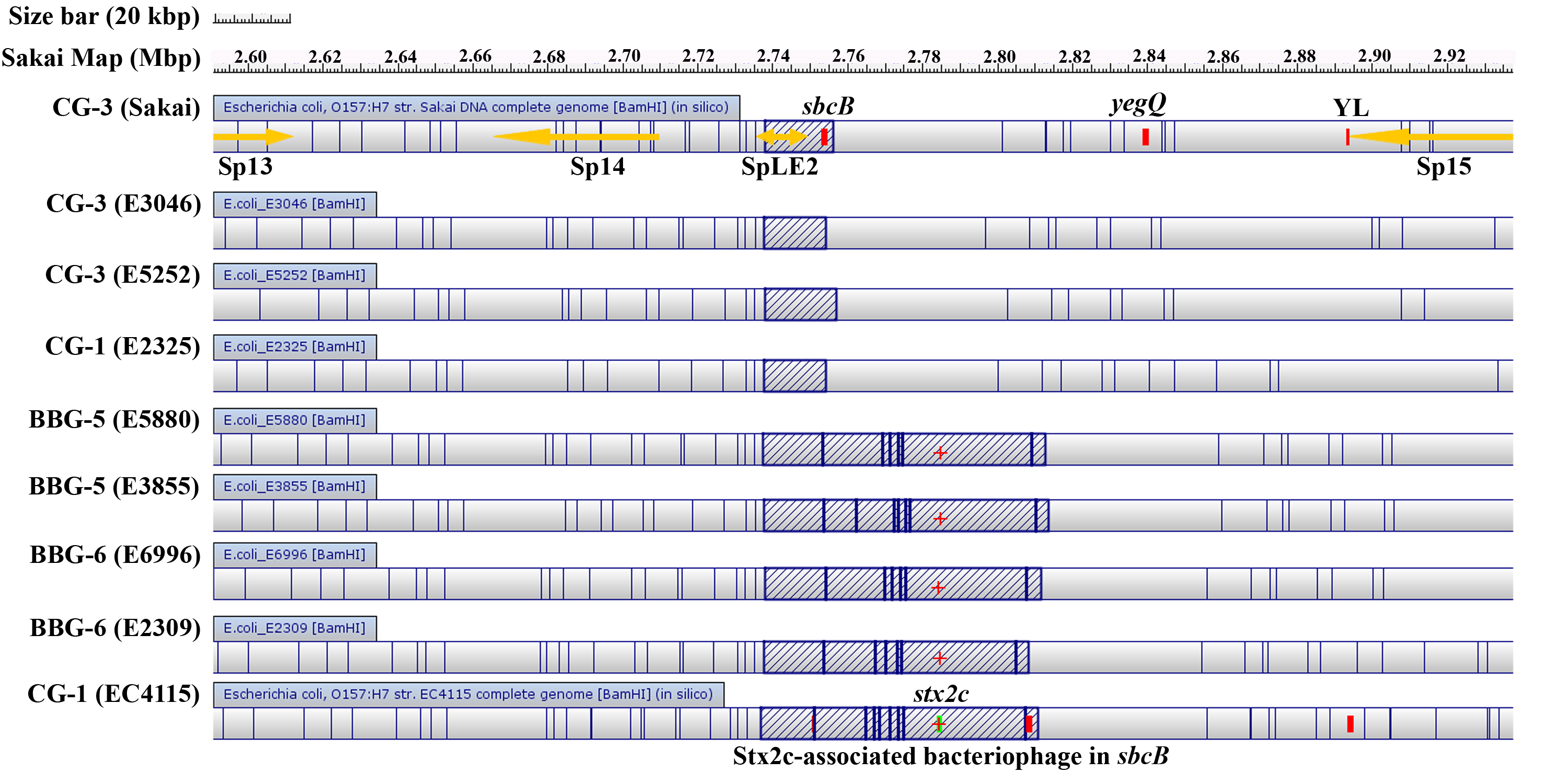

Supplement: Figure S1 — Differences in insertion of Stx2c-associated bacteriophage in sbcB . The differences in insertion of Stx2c-associated bacteriophage in sbcB are shown by the hatched fragments. The yellow arrows indicate the phages (Sp) or prophage–like elements (SpLE) in sequenced strain Sakai (names shown below the map). Red marks indicate the insertion sites (sbcB and yegQ) for Stx-associated bacteriophage, and left junction (YL) for Stx1-associated bacteriophage inserted in yehV (names shown above the map). The restriction enzyme map of sequenced strain EC4115 (GenBank accession # CP001164) shows the known insertion of Stx2c-associated bacteriophage in sbcB locus (hatched fragments). The restriction enzyme map for Sakai and EC4115 are in silico maps and the other seven maps are optical maps of test strains. Plus (+) and minus (−) signs represent presence and absence of stx gene in the strains. (TIF) [file pone.0051572.s001.tif]

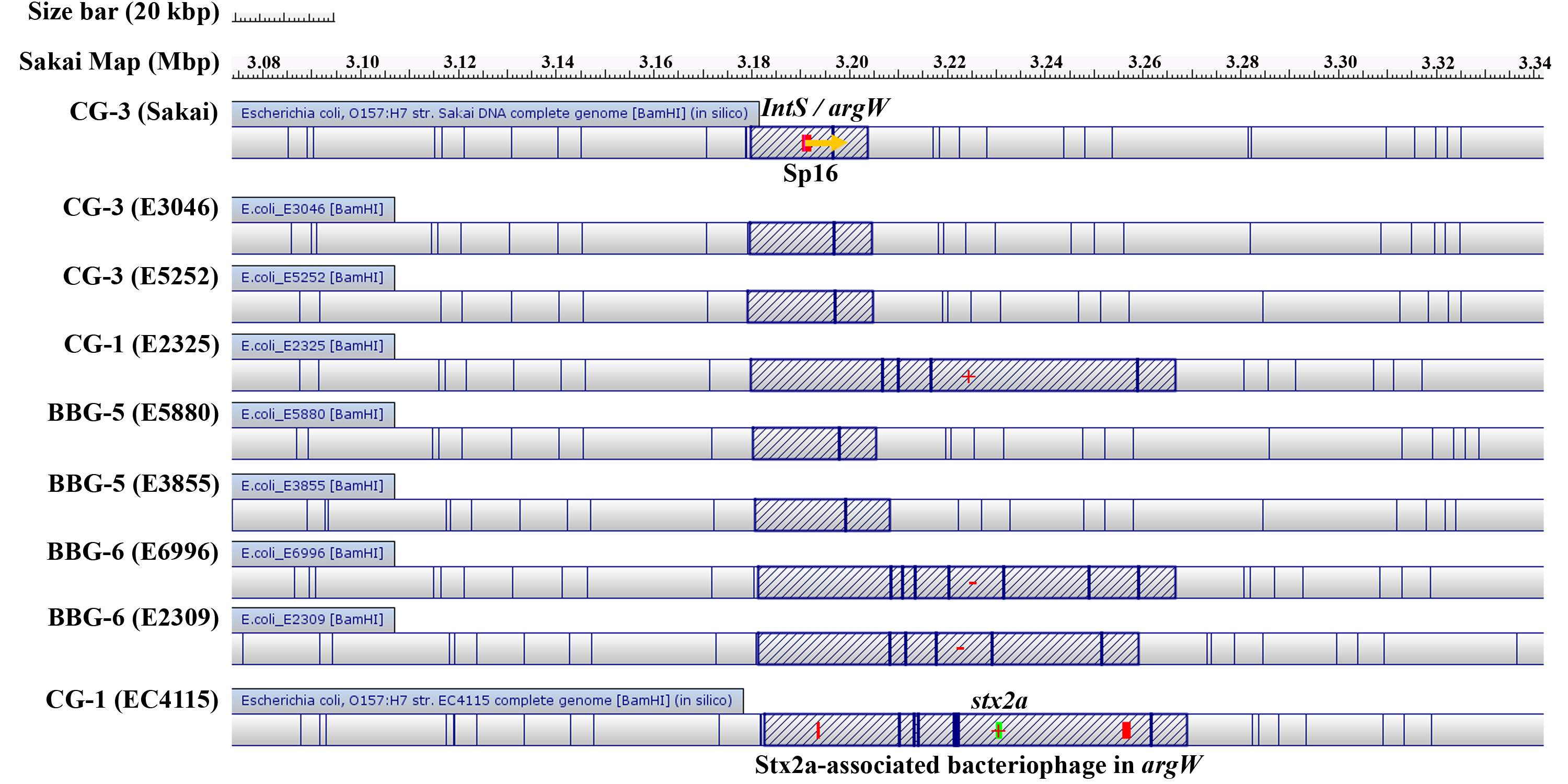

Supplement: Figure S2 — Differences in insertion of Stx2a-associated bacteriophage in argW . The differences in insertion of Stx2a-associated bacteriophage in argW are shown by the hatched fragments. The yellow arrows indicate the phages (Sp) in sequenced strain Sakai (names shown below the map). Red marks indicate the insertion sites (argW/IntS) for Stx-associated bacteriophage (names shown above the map). The restriction enzyme map of the sequenced strain EC4115 (GenBank accession # CP001164) shows the known insertion Stx2a-associated bacteriophage in argW locus (hatched fragments). The restriction enzyme map for Sakai and EC4115 are in silico maps and the other seven maps are optical maps of test strains. Plus (+) and minus (−) signs represent presence and absence of stx gene in the strains. (TIF) [file pone.0051572.s002.tif]
