# Supplementary material for: Carriage of stx2a Differentiates Clinical and Bovine-Biased Strains of Escherichia coli O157
Source: PLoS One. 2012 Dec 11;7(12):e51572. doi: 10.1371/journal.pone.0051572 (PMC3519850; doi:10.1371/journal.pone.0051572)
Supplement: Table S1 — Polymorphic sites in optical maps of seven EHEC O157 strains when compared to the in silico Bam HI restriction enzyme map of the EHEC O157 Sakai strain (CG-3). (DOCX) [file pone.0051572.s003.docx]

**Table S1.** Polymorphic sites in optical maps of seven EHEC O157 strains when compared to the *in silico Bam*HI restriction enzyme map of the EHEC O157 Sakai strain (CG-3).

| S.No. | Sakai Strain | | | | | Polymorphism | | |
| --- | --- | --- | --- | --- | --- | --- | --- | --- |
|  | Start^a^ | End^a^ | *Bam*HI Fragment | Bacteriophages^b^ | S-loops^c^ | Strains^d^ | Type^e^ | Size Difference |
| 1 | 1,46,976 | 1,79,629 | 18 |  | 8 to 11 | 3, 7 | RFLP | 2,039 |
| 2 | 2,14,797 | 2,34,585 | 23-25 |  | None | 1 | RFLP | 236 |
| 3 | 2,66,081 | 2,80,784 | 33 |  | 14 | 1, 6 | RFLP | 2,635 |
| 4 | 2,66,081 | 2,80,784 | 33 |  | 14 | 4,7 | RFLP | 4,007 |
| 5 | 2,66,081 | 2,83,648 | 33-34 |  | 14 | 5 | RFLP | 304 |
| 6 | 2,86,943 | 3,12,150 | 38-39 | Sp1-Sp2 | 16 | 5 | INS | 32,066 |
| 7 | 3,02,183 | 3,12,150 | 39 | Sp1-Sp2 | 16 | 6 | DEL | 9,967 |
| 8 | 3,02,183 | 3,12,150 | 39 | Sp1-Sp2 | 16 | 1, 4, 7 | RFLP |  |
| 9 | 3,02,183 | 3,39,051 | 39-40 | Sp1-Sp2 | 16 and 17 | 5 | RFLP | 7,628 |
| 10 | 3,02,183 | 3,39,051 | 39-40 | Sp2 | 16 and 17 | 4, 7 | INS | 32,000 |
| 11 | 3,12,150 | 3,39,051 | 40 | Sp2 | 16 and 17 | 6 | RFLP | 7,395 |
| 12 | 3,47,657 | 3,66,808 | 43 |  | 18 and 22 | 5 | RFLP | 2,720 |
| 13 | 4,02,991 | 4,24,456 | 47-48 |  | 28 to 30 | 2 | RFLP | 695 |
| 14 | 5,41,606 | 6,08,995 | 59 |  | 40 to 43 | 1, 3, 5, 7 | RFLP | 5,729 |
| 15 | 6,82,754 | 6,97,079 | 69 |  | 45 | 4 | RFLP | 1,630 |
| 16 | 8,91,357 | 9,14,056 | 90-95 | Sp3 | 56 | 5 | DEL | 22,699 |
| 17 | 9,14,056 | 9,39,948 | 96-97 |  | None | 5 | INS | 11,999 |
| 18 | 1,186,975 | 1,189,684 | 133 | Sp4 | 67 | 2, 6 | DEL | 2,709 |
| 19 | 1,223,450 | 1,230,921 | 139-140 |  | None | 1 | RFLP | 130 |
| 20 | 1,246,750 | 1,277,114 | 142-145 | Sp5 | 69 | 3 | RFLP | 199 |
| 21 | 1,287,972 | 1,310,488 | 150-151 | Sp5 | 69 | 3 | RFLP | 239 |
| 22 | 1,246,750 | 1,310,488 | 142-151 | Sp5 (*wrbA*) | 69 | 1, 4, 5, 6, 7 | DEL | 63,738 |
| 23 | 1,341,342 | 1,365,042 | 154-155 |  | 71 | 1 | RFLP | 1,052 |
| 24 | 1,341,342 | 1,377,110 | 154-156 | SpLE 1 | 71 & 72 | 6 | RFLP | 226 |
| 25 | 1,362,712 | 1,377,110 | 155-156 | SpLE 1 | 71 & 72 | 5 | INS | 36,091 |
| 26 | 1,377,110 | 1,409,739 | 157-161 | SpLE 1 | 72 | 5 | DEL | 32,620 |
| 27 | 1,407,326 | 1,409,739 | 161 | SpLE 1 | 72 | 1, 2 | DEL | 2,413 |
| 28 | 1,412,582 | 1,458,219 | 163 | SpLE 1 | 72 | 3, 4, 7 | RFLP | 11,339 |
| 29 | 1,540,236 | 1,549,117 | 171--172 | Sp6 | 77 | 3 | RFLP | 407 |
| 30 | 1,565,532 | 1,567,790 | 177 | Sp6 | 77 | 5, 6 | DEL | 2,258 |
| 31 | 1,567,790 | 1,570,529 | 178 | Sp6 | 77 | 3, 5 | RFLP |  |
| 32 | 1,572,018 | 1,609,079 | 180-181 | Sp6/7 | 77 & 78 | 1, 4, 5, 7 | RFLP | 3,262 |
| 33 | 1,572,018 | 1,782,527 | 180-207 | Sp6/7/8/9 | 77 to 85 | 6 | TINV |  |
| 34 | 1,652,804 | 1,673,856 | 186-187 | Sp8 | 79 | 5 | INS | 16,591 |
| 35 | 1,673,856 | 1,696,818 | 188-190 |  | 80 to 83 | 4, 7 | RFLP | 25,000 |
| 36 | 1,698,127 | 1,708,267 | 192-193 |  | 84 | 1 | RFLP | 2,100 |
| 37 | 1,699,688 | 1,708,267 | 193 |  | 84 | 4, 7 | DEL | 8,579 |
| 38 | 1,784,006 | 1,786,264 | 210 | Sp9 | 85 | 6, 7 | DEL | 2,258 |
| 39 | 1,786,264 | 1,838,526 | 211-218 | Sp9 | 85 & 86 | 4 | SUB | 14,505 |
| 40 | 1,790,493 | 1,834,215 | 214-216 | Sp9 | 85 & 86 | 7 | RFLP | 2,106 |
| 41 | 1,790,493 | 1,807,306 | 214-215 | Sp9 | 85 | 3 | INS | 43,755 |
| 42 | 1,802,604 | 1,834,215 | 215-216 | Sp9 | 85 & 86 | 3 | RFLP | 1,329 |
| 43 | 1,885,324 | 1,902,729 | 221-222 |  | 88 & 89 | 1, 2, 5 | RFLP | 707 |
| 44 | 1,838,526 | 1,906,566 | 219-224 |  | 87 to 89 | 4 | INV | 68,040 |
| 45 | 1,904,726 | 1,957,800 | 224-235 | Sp10 | 90 to 93 | 1, 4, 5, 7 | DEL | 53,074 |
| 46 | 1,942,493 | 2,242,093 | 230-275 | Sp10/11/12 | 93 to 108 | 1 | INV | 2,99,600 |
| 47 | 1,956,301 | 2,223,298 | 230-275 | Sp10/11/12 | 93 to 108 | 3 | INV | 2,66,997 |
| 48 | 1,951,304 | 2,227,543 | 230-275 | Sp10/11/12 | 93 to 108 | 6 | INV | 2,76,239 |
| 49 | 1,957,800 | 2,191,550 | 230-275 | Sp10/11 | 93 to 108 | 4 | INV | 2,33,750 |
| 50 | 1,957,800 | 1,977,340 | 236 | Sp10 | 93 | 1, 5, 7 | RFLP | 296 |
| 51 | 1,998,259 | 2,005,973 | 239-240 |  | 98 | 1 | RFLP | 986 |
| 52 | 2,118,229 | 2,123,573 | 253 |  | None | 5 | RFLP | 3,001 |
| 53 | 2,157,343 | 2,227,543 | 257-269 | Sp11/12 | 108 | 1 | RFLP |  |
| 54 | 2,188,160 | 2,191,550 | 262 | Sp 11 | 108 | 7 | DEL | 3,390 |
| 55 | 2,191,550 | 2,221,208 | 263-265 | Sp 11/12 | 108 | 4 | SUB | 15,545 |
| 56 | 2,192,091 | 2,223,298 | 264-266 |  | 108 | 1, 5, 7 | RFLP |  |
| 57 | 2,208,818 | 2,221,208 | 265 | Sp12 | 108 | 7 | INS | 14,721 |
| 58 | 2,223,298 | 2,227,543 | 267-269 | Sp12 | 108 | 1, 4, 7 | RFLP |  |
| 59 | 2,227,543 | 2,229,801 | 270 | Sp12 | 108 | 1, 5 | DEL | 2258 |
| 60 | 2,242,093 | 2,252,868 | 276 | Sp12 | 108 & 109 | 3, 4, 5, 7 | RFLP | 1,000 |
| 61 | 2,257,438 | 2,259,545 | 279 |  | None | 5 | DEL | 2,107 |
| 62 | 2,351,630 | 2,387,477 | 291 |  | None | 3 | RFLP | 2,533 |
| 63 | 2,435,134 | 2,496,501 | 297-298 |  | 115 to 117 | 2, 3, 6 | RFLP | 4,142 |
| 64 | 2,599,467 | 2,619,557 | 311-312 | Sp13 | 119 | 2 | DEL | 20,090 |
| 65 | 2,686,383 | 2,696,176 | 321-322 | Sp 14 | 125 | 6 | INS | 2,644 |
| 66 | 2,696,176 | 2,706,692 | 323-324 | Sp 14 | 125 | 5, 7 | RFLP |  |
| 67 | 2,706,692 | 2,709,729 | 325 | Sp 14 | 125 | 1 | RFLP |  |
| 68 | 2,740,136 | 2,758,421 | 334 | SpLE 2 | 129 | 1, 3 | RFLP |  |
| 69 | 2,758,421 | 2,803,536 | 335 | SpLE 2 (*sbcB*) | 130 to 134 | 3 | RFLP | 2,361 |
| 70 | 2,740,136 | 2,803,536 | 334-335 | SpLE 2 (*sbcB*) | 130 to 134 | 4, 5, 6, 7 | INS | 60,000 |
| 71 | 2,846,209 | 2,846,932 | 343 | *yegQ* | None | 1, 6 | SUB | 31,502 |
| 72 | 2,849,467 | 2,910,044 | 345 | Sp15 | 138 to 153 | 1, 3, 4, 7 | RFLP | 2,000 |
| 73 | 2,910,044 | 2,912,120 | 346 | Sp15 | 153 | 1, 2 | DEL | 2,076 |
| 74 | 2,912,120 | 2,943,884 | 347-349 | Sp15 (*stx1*) | 153 | 4, 5 | SUB |  |
| 75 | 2,918,479 | 2,943,884 | 349 | Sp15 (*stx1)* | 153 | 6, 7 | RFLP | 2,405 |
| 76 | 3,025,384 | 3,027,728 | 360 |  | None | 4 | DEL | 2,344 |
| 77 | 3,181,098 | 3,205,898 | 379-381 | Sp16 | 168 to 172 | 5 | RFLP | 2,918 |
| 78 | 3,181,908 | 3,205,898 | 380-381 | Sp16 (*argW & IntS*) | 169 to 172 | 7 | RFLP | 3,441 |
| 79 | 3,181,908 | 3,198,717 | 380 | Sp16 (*argW & IntS*) | 169 to 172 | 1, 4, 6 | RFLP | 9,810 |
| 80 | 3,180,971 | 3,181,908 | 378-379 | Sp16 | 168 to 179 | 6 | INS | 48,365 |
| 81 | 3,198,717 | 3,219,331 | 381-382 | Sp16 | 172 | 1, 7 | INS | 60,000 |
| 82 | 3,322,135 | 3,324,466 | 395 |  | None | 2 | DEL | 2,331 |
| 83 | 3,324,466 | 3,327,259 | 396 |  | 176 | 5 | DEL | 2,793 |
| 84 | 3,423,621 | 3,452,513 | 409-412 |  | 182 to 184 | 1 | RFLP |  |
| 85 | 3,462,832 | 3,465,501 | 417 |  | None | 2, 4 | DEL | 2,669 |
| 86 | 3,470,603 | 3,479,545 | 420 | Sp17 (*ssrA)* | 186 | 1, 2 | RFLP |  |
| 87 | 3,479,545 | 3,489,849 | 421-423 | Sp17 | 186 | 5, 6 | DEL / RFLP |  |
| 88 | 3,489,849 | 3,501,289 | 424 | Sp17 | 186 | 1, 5 | RFLP | 1,953 |
| 89 | 3,599,625 | 3,622,254 | 435-436 |  | 192 | 5 | RFLP | 3,076 |
| 90 | 3,605,371 | 3,622,254 | 436 |  | 192 | 4 | RFLP | 1,181 |
| 91 | 3,633,688 | 3,658,281 | 439-440 |  | 195 | 2 | RFLP | 211 |
| 92 | 3,799,506 | 3,813,159 | 457-458 |  | 202 | 2 | RFLP |  |
| 93 | 3,945,664 | 3,963,810 | 480-482 |  | None | 1 | RFLP |  |
| 94 | 3,985,602 | 4,023,596 | 489 |  | 211 | 3, 6 | RFLP |  |
| 95 | 4,081,768 | 4,090,041 | 495-496 |  | None | 1, 6 | RFLP |  |
| 96 | 4,351,588 | 4,411,722 | 529 |  | 226 to 231 | 3, 7 | RFLP | 2,511 |
| 97 | 4,559,403 | 4,566,073 | 541 |  | 242 | 1 | DEL | 6,670 |
| 98 | 4,559,403 | 4,566,073 | 541 |  | 242 | 7 | SUB | 13,105 |
| 99 | 4,566,073 | 4,599,946 | 542-543 | SpLE4 | 244 | 1 | INS | 21,511 |
| 100 | 4,599,946 | 4,624,256 | 544-545 | SpLE4 | 244 | 2 | RFLP |  |
| 101 | 4,719,258 | 4,721,357 | 558 |  | None | 4, 5 | DEL | 2,099 |
| 102 | 4,792,336 | 4,830,632 | 566 |  | 256 to 258 | 7 | SUB | 35,627 |
| 103 | 4,882,079 | 4,895,291 | 575-576 |  | 265 | 1 | RFLP |  |
| 104 | 5,025,260 | 5,084,627 | 593 | Sp18 | 274 | 1, 2, 3, 4, 5, 6, 7 | SUB | 38,000 |
| 105 | 5,106,338 | 5,108,460 | 596 | Sp18 | None | 2 | DEL | 2,122 |
| 106 | 5,108,460 | 5,145,333 | 597-598 | Sp18 | 275 | 4, 5 | SUB | 9,000 |
| 107 | 5,108,460 | 5,148,412 | 597-599 | Sp18 | 275 | 1, 6 | SUB | 7,000 |
| 108 | 5,110,111 | 5,145,333 | 598 | Sp18 | 275 | 7 | SUB | 7,000 |
| 109 | 5,160,490 | 5,184,052 | 602 | Sp18 | 276 | 6 | RFLP |  |
| 110 | 5,249,591 | 5,266,494 | 610-611 |  | None | 6 | RFLP |  |
| 111 | 5,267,849 | 5,269,872 | 614 |  | None | 5 | DEL | 2,023 |
| 112 | 5,341,907 | 5,361,383 | 621 | SpLE 5/6 | 285 to 287 | 4, 7 | RFLP | 2,000 |

^a^ Chromosomal locations of polymorphisms in Sakai genome.

^b^ Parenthesis includes genes present in the specific region; Sp = Sakai prophage; SpLE = Sakai prophage like elements

^c^ S-loops are the Sakai genomic specific segments scattered across the common genomic backbone shared with *Escherichia coli* K-12 strain.

^d^ Strains affected (1=E2325, 2=E5252, 3=E3046, 4=E5880, 5=3855, 6=2309, 7=E6996)

^e^ Genetic rearrangements in comparison with strain Sakai (CG-3). Insertions (INS)= presence of additional RE fragments; deletions (DEL) = loss of RE fragments; restriction fragment length polymorphisms (RFLPs) = altered sizes and/or numbers of RE fragments; inversions (INV) = RE fragments in reversed order; substitutions (SUB) = altered RE fragments; transversion (TNV) = RE fragments in reversed order and in a different location.
